# Ear Asymmetry and Contextual Influences on Speech Perception in Hearing-Impaired Patients

**DOI:** 10.3389/fnins.2022.801699

**Published:** 2022-03-18

**Authors:** Annie Moulin

**Affiliations:** Perception, Attention and Memory Team (PAM), Lyon Neuroscience Research Center, INSERM U1028 – CNRS UMR 5292, University of Lyon, Lyon, France

**Keywords:** hemispheric asymmetry, contextual influences, speech perception, hearing-loss, laterality, ageing

## Abstract

The left hemisphere preference for verbal stimuli is well known, with a right ear (RE) advantage obtained when competing verbal stimuli are presented simultaneously, at comfortable intensities, to both ears. Speech perception involves not only the processing of acoustic peripheral information but also top–down contextual influences, filling the gaps in the incoming information that is particularly degraded in hearing-impaired individuals. This study aimed to analyze the potential asymmetry of those contextual influences on a simple speech perception task in hearing-impaired patients in light of hemispheric asymmetry. Contextual influences on disyllabic word perception scores of 60 hearing-impaired patients were compared between left ear (LE) and RE, in a balanced design, involving two repetitions of the same task. Results showed a significantly greater contextual influence on the RE *versus* the LE and, for the second repetition *versus* the first one, without any interaction between the two. Furthermore, the difference in contextual influences between RE and LE increased significantly with the RE advantage measured by a dichotic listening test in the absence of any significant correlation with hearing threshold asymmetry. Lastly, the contextual influence asymmetry decreased significantly as age increased, which was mainly due to a greater increase, with age, of contextual influences on the LE *versus* the RE. Those results agree with the literature reporting a relative right-shift of hemispheric asymmetry observed with age in speech in noise perception tasks in normal hearing subjects and the clinical reports of generally better audiometric speech scores obtained in RE *versus* LE.

## Introduction

Since the seminal study of Kimura (1961), the left hemisphere’s preference for verbal stimuli processing is well known, with a right ear advantage (REA) obtained when two competing verbal auditory stimuli are presented simultaneously, at comfortable levels, to both ears, in a dichotic listening task (DLT): individuals preferably repeat words presented in the right ear (RE) *versus* words presented in the left ear (LE). This REA is taken as an index of hemispheric asymmetry for language processing and depends, as well, on auditory attention, e.g., the ability to focus on one ear while inhibiting the processing of information of the other ear (Kinsbourne, 1970; Hiscock and Kinsbourne, 2011). Independently testing both ears allows to take out the competition between both inputs and still yields an REA (Geffen and Quinn, 1984; Hiscock and Kinsbourne, 2011). A REA has been obtained in several clinical studies that have compared the consequences of single-sided deafness on the RE *versus* the LE on speech perception scores, which were lower in the healthy LE than in the healthy RE (Wettstein and Probst, 2018). Similar findings were obtained in children, in a more challenging speech in noise perception task, with poorer scores on the LE *versus* the RE (Hartvig Jensen et al., 1989). Similarly, in unilateral cochlear implanted patients, RE cochlear implants seemed to give better outcomes than LE cochlear implants (Henkin et al., 2008). However, as those studies necessarily compared RE and LE from different patients’ groups, it is difficult to rule out minor group differences in cognitive aspects, auditory attention, or hearing damage on the remaining ear. Speech perception does not involve only the processing of acoustic peripheral information but also top–down mechanisms filling the gaps in the incoming information to obtain a meaningful percept (e.g., Davis and Johnsrude, 2007; Pichora-Fuller, 2008). Those top–down influences, or contextual influences (CI), are particularly important in hearing-impaired patients, in whom the peripheral auditory information is partially lacking, often associated with impaired auditory processing in both the temporal (Gordon-Salant et al., 2011) and frequency domains (Phillips et al., 2000). The present study aimed to compare CI on disyllabic word perception on the RE *versus* the LE of hearing-impaired patients and to see if this potential CI asymmetry could be related to hemispheric asymmetry as assessed by a DLT.

## Materials and Methods

### Protocol

Sixty (31 women) French-native hearing-impaired patients aged 69.3 years [standard deviation (SD) = 13] participated in the study. A more detailed description of the population is available later and as Supplementary Material.

#### Cognitive and Hearing-Abilities Screening

Tonal and speech audiometry were performed in a sound-proof booth, using an Interacoustics Callisto audiometer, and the first ear tested was counterbalanced across participants.

Tonal audiometry was performed at octave frequencies ranging from 250 to 8 kHz. The mean hearing threshold (HT) on the better ear was 33.3 dB HL (SD = 12.3). The average RE HT was 36.6 dB HL (SD = 12.7) with 36.5 dB (SD = 13.8) for the LE. Whatever the audiometric frequency considered, no statistically significant differences between RE and LE HT were found [repeated-measures analysis of variance (RAnova), *F*(1,58) = 0.7, p = ns]. The average HT, both ears combined, ranged from 25 dB HL at 250 Hz up to 70 dB HL at 8 kHz.

Speech audiometry was performed for each ear using the standard linguistic material used in audiological practice in France, i.e., Fournier’s disyllabic word lists (Fournier, 1951). This allowed to define, for each ear, the speech reception threshold (SRT) [39 dB sound pressure level (SPL) (SD = 14.2) for the RE *versus* LE 38.1 dBSPL (SD = 13.8)] and the minimum intensity yielding 100% correct speech scores (100% speech threshold) [54.4 dBSPL (SD = 14.8) for the RE *versus* LE 54.3 dBSPL (SD = 13.9)].

The cognitive screening was performed using the Montreal Cognitive Assessment (MOCA) test (Nasreddine et al., 2005) and a French-language lexical test (Deltour, 1998).

#### Laterality Behavioral Tests

The patient’s handedness index (HI) was calculated from the Edinburgh handedness questionnaire (Oldfield, 1971).

A validated French-language DLT (Demanez and Demanez, 2011) was performed at an intensity of at least 6 dB above the 100% speech threshold of the worse ear, using two blocks of 12 trials, each trial composed of two pairs of two monosyllabic words presented simultaneously in each ear (detailed in the Supplementary Material). A free recall paradigm was used.

#### Assessment of Contextual Influences on Speech Perception

To assess CI on word perception, an open-set speech perception test was used, using 60 words from French disyllabic word lists, presented at the same intensity for both ears, adapted for each patient, so that this intensity yielded between 30 and 70% perception scores on each ear. The 60 words were specifically chosen, from the extensive Fournier disyllabic word lists, so that they were different from the word lists usually presented to patients in French audiology clinics.

**FIGURE 1:**
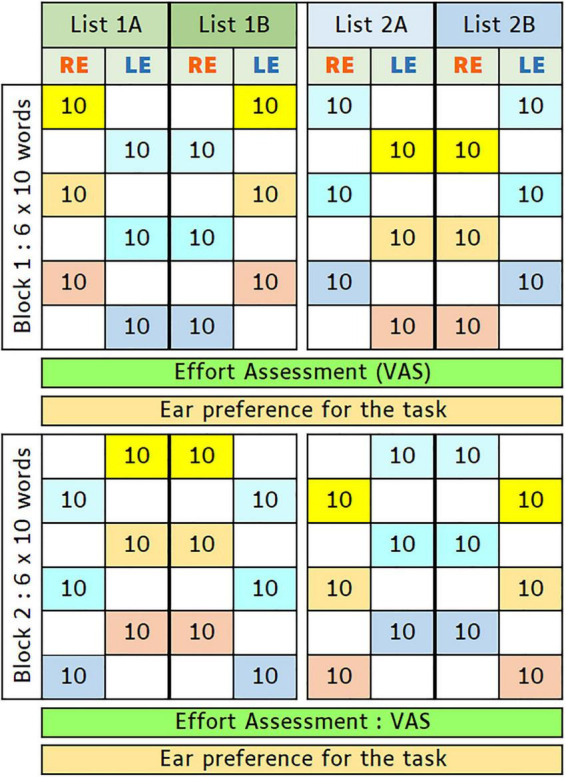
Protocol used in the main speech perception task: 6 sets of 10 words (each set represented by a colored cell with number 10) were alternatively presented to left (LE) and right ears (RE) of a participant (block 1). After a pause, in which the participant was invited to answer two questions (about listening effort and the ear in which the task was the easiest), the same 6 sets of 10 words were presented (block 2), with inverted LE and RE channels (e.g., list 1A). Matching list 1B was used for another group of participants. Another pair of word lists (2A and 2B), differing by order of the word sets, was used for two other participant groups. Assignment of each word list to a participant was by order of inclusion in study, and each word list was used for 15 participants (i.e., for a total of 60 participants).

A stereo sound file (wav format, a sampling frequency of 44.1 kHz) was constructed using the sound files provided as the usual material for speech audiometry so that the 60 words were presented alternatively to the LE and RE, at the same intensity, per set of 10 words (Figure 1). Each word onset was separated from the next word onset by 4.5 s, allowing the participant to repeat the word heard, regardless on which ear it was presented. The experimenter who scored the words pronounced by the participant did not know whether the words were presented to the LE or the RE of the participant. The scores were obtained in words, syllables, and phonemes.

A second matching sound file, with left and right side inverted, was presented to the participant after a 5-min pause, in a second block, so that each one of the 60 words was presented once to the RE and once to the LE at the same level for each participant. The order of those lists was counterbalanced so that half the participants started by the RE (one list) and half by the LE (the second list).

Another pair of two matching lists were built, differing from the first pair by order of the 10-word sets within each block. Hence, a total of four combinations were used to counterbalance the first ear tested (RE or LE), the word order (two different word orders), and the effects of learning and memory (blocks 1 and 2). Each patient was attributed one of the four combinations, in the order of the inclusion in the study: this resulted in each combination used for 15 participants, for a total of 60 participants tested. *A posteriori* checks were made to be sure that no significant differences in participants’ characteristics (age, sex, and hearing thresholds) were obtained according to the pairs of combinations used.

After each block of 60 words, the participants were asked to evaluate, on an 11-point visual analog scale, the amount of effort they felt they had to exert to perform the task (from 0: no effort at all, up to 10: maximum effort). With a second scale, they were asked in which ear the task was perceived as easier (from −5: definitely easier on the LE, to +5: definitely easier on the RE).

### Statistical Analysis

#### Laterality Indexes

Handedness was assessed using the HI, defined as HI = 100 × (RH – LH)/(RH + LH), ranging from −100 to 100 [with RH (LH) the number of points, from 0 to 2, given for right hand (left hand) preference]. Forty-nine participants showed an HI of 100, and two participants were left-handers (HI of −100).

The number of words correctly repeated for the DLT was counted separately for each side (LE or RE) and combined in the DLT speech score, which was in the normative range for 65- to 74-year-olds (mean = 30.4%, SD = 11.4). The REA was calculated using the laterality index (LI) formula:


$$LI=\frac{\left(REwordscore-LEwordscore\right)*100}{Numberofwordsrepeated}$$

HT asymmetry was calculated as the LE–RE difference in HT averaged across all octave frequencies. Similarly, SRT and 100% speech score asymmetries were calculated as the LE–RE difference in SRT (and 100% speech score thresholds), so that a positive difference meant better RE thresholds.

#### Contextual Influences

Speech perception scores were obtained for both 60-word blocks and both ears of each participant. CI was measured using Boothroyd and Nittrouer’s (1988) j factor, according to Eq. 1.


$$j=\frac{\text{log}\left(phonemicpercentscore\right)}{\text{log}\left(wordpercentscore\right)}$$

A j factor of 1 means that one part is sufficient for the entire word recognition, i.e., contextual influence is maximum. This index of CI was calculated, as well, using the phonemic and syllabic scores (CI*pho_syll*) and using the syllabic and word scores CI*syll_word*. As the j factor does not follow a normal distribution, statistics were performed on an inverse cubic transformation (j^–3^), with CI increasing as j^–3^ increases, up to the maximum of 1. The CI asymmetry was calculated as the difference between RE and LE CI. As there was a very good correlation between CI*syll_word* and CI*pho_word* (*r* = 0.92, *p* < 0.0001), and as both CI indexes yielded very similar results, only the CI*pho_word* is presented in the manuscript, and the results concerning CI*syll_word* and CI*phon_syll* are presented in the Supplementary Material.

#### Statistical Tests

Correlations were performed per pair of variables, and *p*-values were adjusted (mentioned as *p*_*adj*_) for multiple comparisons, using the Benjamini and Hochberg procedure (Benjamini and Hochberg, 1995) with a false discovery rate of 0.05. As bivariate normality was not always met for each pair, as assessed by Shapiro–Wilk tests, Spearman’s rank correlations (rho) are reported when relevant. Differences between correlation coefficients were assessed using Fisher’s z transformation (Steiger, 1980). For RAnova, the Greenhouse–Geisser correction for sphericity was applied when relevant. Bonferroni’s correction for multiple tests was applied to *post-hoc* tests (mentioned as p_bonf_).

## Results

### Speech Perception Scores and Contextual Influences

**FIGURE 2:**
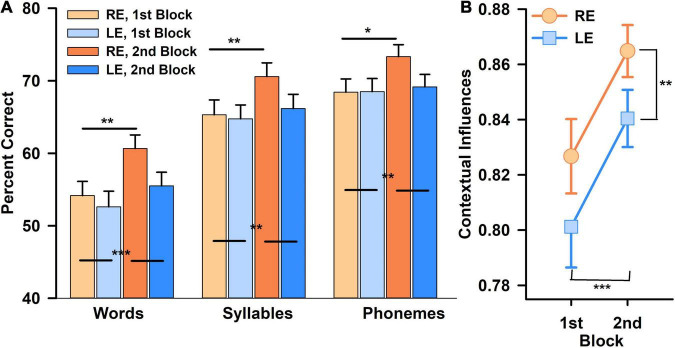
Percent correct scores obtained in speech perception task (left panel), for the right ear (RE, orange) and the left ear (LE, blue), for each block (first block in light colors, second block in darker colors). Percent scores were calculated in words, syllables, and phonemes **(A)**. Contextual influences (j^–3^) obtained for RE (orange) and LE (blue) for each block. The closer the index (j^−3^) is to 1, the greater the contextual influences are **(B)**. Statistically significant differences are mentioned as stars after correction for multiple tests (*p < 0.05; **p < 0.01; ***p < 0.001).

An increase in word scores between blocks 1 and 2 was observed. A three within-subjects RAnova (type of score × block × ear) showed a highly significant type of score effect, [*F*(1.7,103) = 235, *p* < 0.00001, η_*p*_ = 0.80, ω^2^ = 0.22], with greater phonemes scores than word scores; block effect [*F*(1,59) = 13, *p* < 0.00001, η_*p*_ = 0.19, ω^2^ = 0.022] with greater scores for the second block (Figure 2A), but no overall significant ear effect [*F*(1,59) = 2.2, *p* = ns]. However, there was a significant interaction between type of score and ear [*F*(1.67,98) = 5.4, *p* < 0.01, η_*p*_ = 0.08], and between type of score and block, with greater increase of word scores than phoneme scores, from blocks 1 to 2 [*F*(1.4,83) = 11.9, *p* < 0.00001, η_*p*_ = 0.04, ω^2^ = 0.004]. The three-way interaction (score type × block × ear) was not statistically significant. For the second block, RE scores tended to be greater than LE scores (*p* < 0.05), but it did not reach statistical significance after correction for multiple tests.

A two within-subjects (ear × block) RAnova showed a significantly greater CI*pho_word* on the RE *versus* the LE [*F*(1,59) = 8.8, *p* < 0.005, η_*p*_ = 0.13, ω^2^ = 0.02] and for the second block *versus* the first one [*F*(1,59) = 26.1, *p* < 0.00001, η_*p*_ = 0.31, ω^2^ = 0.053], without any significant interaction between the two (Figure 2B). *Post-hoc* tests confirmed a significantly greater CI in the RE *versus* the LE (*t* = 2.97, *p*_*bonf*_ < 0.004, Cohen’s *d* = 0.38) and a significantly greater CI for the second block than the first one (*t* = 5.1, *p*_*bonf*_ < 0.00001, Cohen’s *d* = 0.66) (Figure 2B). HT asymmetry, used as a covariate, did not show any significant effect with any of those results, nor did the type of word list used. However, the LI, used as covariate, showed a significant effect with the ear: *F*(1,58) = 4.6, *p* < 0.04, η_*p*_ = 0.073, ω^2^ = 0.009, and a tendency for an interaction with ear and block [*F*(1,58) = 3.6, *p* = 0.06].

### Contextual Influences Asymmetry and Laterality Indexes

**FIGURE 3:**
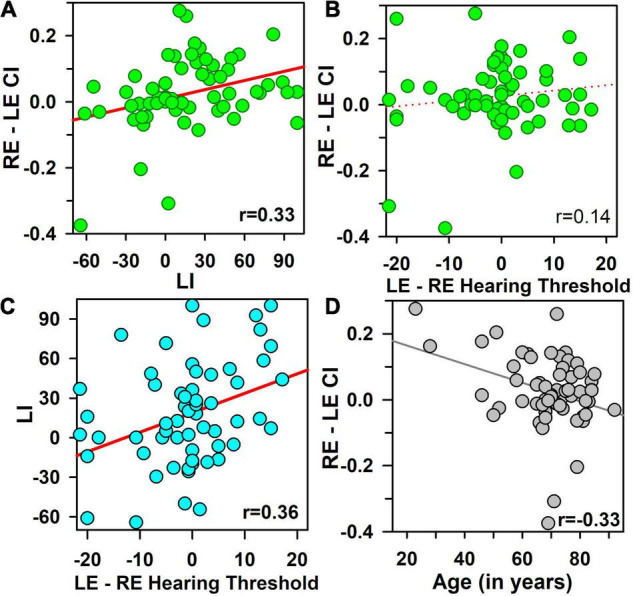
Contextual influence asymmetry [calculated as the difference between right and left ears (RE–LE CI)], as a function of the laterality index [LI, obtained in a dichotic listening task, **(A)]**, and as a function of hearing threshold asymmetry [LE–RE hearing threshold, **(B)**]. The Laterality index (LI) is shown as a function of hearing-threshold asymmetry (difference in hearing threshold between LE and RE), **(C)**. Contextual influence asymmetry (RE–LE CI) is shown as a function of age of participants in **(D)**. Pearson’s correlation coefficients are mentioned in each panel, and all coefficients greater than 0.30 (in bold fonts) are statistically significant (*p* < 0.01, *p*_*adj*_ < 0.05).

The CI asymmetry increased significantly with the LI (*r* = 0.33, *p* < 0.01, *p*_*adj*_< 0.04) (Figure 3A), only for the first block (rho = −0.02, *p* = ns for the second block), but not with the HT asymmetry (*r* = 0.14, *p* = ns) (Figure 3B), nor with SRT asymmetry (*r* = 0.07, *p* = ns), nor with 100% speech score threshold asymmetry (*r* = 0.09, *p* = ns).

The LI increased significantly with the HT asymmetry (*r* = 0.36, *p* = 0.005, *p*_*adj*_ = 0.026) (Figure 3C), but not with the SRT asymmetry (*r* = 0.27, *p* < 0.05, *p*_*adj*_ = ns), nor the speech 100% threshold asymmetry (rho = 0.12, *p* = ns) (Table 1). The correlation between CI asymmetry and LI were significantly stronger than the correlations between CI asymmetry and HT asymmetry (*z* = 2.31, *p* < 0.02). No significant correlations were obtained between HI and LI (rho = 0.13, *p* = ns).

**TABLE 1:** Pearson’s correlation coefficients between the main variables of study. Laterality index (LI) has been obtained from dichotic listening task (DLT).

	LI	100% speech threshold asymmetry	HT asymmetry	CI asymmetry	Ear preference	RE CI	LE CI	Percent improvement between blocks	Percent correct DLT	Better ear PTA	Age
LI	—										
100% speech threshold asymmetry	0.19	—									
HT asymmetry	**0.36**	**0.76**	—								
CI Asymmetry	**0.33**	0.09	0.14	—							
Ear preference	0.01	0.02	0.01	***0.28***	—						
RE CI	0.15	0.15	0.22	**0.43**	0.03	—					
LE CI	–0.18	0.06	0.07	**-0.57**	–0.24	**0.50**	—				
Percent improvement between blocks	–0.12	–0.19	–0.15	***0.26***	0.21	–0.23	**-0.46**	—			
Percent correct DLT	–0.14	0.14	0.09	0.05	–0.03	0.11	0.05	0.10	—		
Better ear PTA	0.02	–0.08	0.06	–0.12	0.11	–0.19	–0.05	–0.09	−**0.35**	—	
Age	0.04	0.00	–0.07	−**0.33**	–0.10	–0.04	***0.28***	−**0.38**	−**0.44**	0.25	—

*The 100% speech threshold asymmetry is the difference between LE and RE thresholds for 100% speech perception scores. Similarly, hearing-threshold asymmetry (HT asymmetry) and contextual influence asymmetry for first block (CI asymmetry) have been calculated as the difference between left ear (LE) and right ear (RE). Ear preference indicates in which ear (RE or LE) the task was considered as easier, with positive number for right side (and negative for left side). Contextual influences measures on right ear (RECI) and on left ear (LECI) are present for first block. The improvement in speech scores (word scores) between both blocks is mentioned as “percent improvement between blocks.” Percent correct DLT is the percentage of correct responses (for both right and left ears) obtained in the dichotic listening task. Better ear PTA is the lowest hearing threshold averaged across all tested audiometric frequencies of both ears. P-values have been adjusted (p_adj_) according to the Benjamini and Hochberg procedure (Benjamini and Hochberg, 1995) using a false discovery rate of 0.05. Significant correlation coefficients are mentioned in bold fonts (p_adj_ < 0.05), on a light gray background (p_adj_ < 0.01) or darker gray background (p_adj_< 0.001). Correlation coefficients that were significant only to non-adjusted p-values (p < 0.05) are mentioned in bold italic fonts.*

A multi-regression model showed that the CI asymmetry was explained significantly by the LI (β = 0.34, *p* = 0.005) and age (β = −0.34, *p* = 0.005) [*r*^2^= 0.22, *F*(2,57) = 8.2, *p* < 0.001]: it increased with LI and decreased with age. None of the other tested variables (in particular HT asymmetry, cognitive tests, handedness, and HT) reached statistical significance.

### Self-Assessments of Listening Effort and Ear Preference

Both the self-assessed listening effort (*r* = 0.77, *p* < 0.00001, *p*_*adj*_< 0.00001) and the self-assessed ear preference (*r* = 0.66, *p* < 0.00001, *p*_*adj*_< 0.00001) showed strong correlations between the first and second blocks. However, the listening effort increased significantly in the second block (6.33, SD = 2.33, *versus* the first block: 5.83, SD = 2.03, *w* = 192, *p* < 0.01), whereas the ear preference did not change significantly (0.62 *versus* 0.63, *W* = 146, *p* = ns), showing, for both blocks, a small preference for the RE.

Correlations were observed between the self-assessed ear preference and the CI asymmetry, with the task labeled as easier on the RE, as the CI asymmetry was shifting toward the RE (rho = 0.29, *p* < 0.03, *p*_*adj*_ = ns for the 1st block and rho = 0.38, *p* = 0.003, *p*_*adj*_ = 0.04) for the second block. The ear preference shifted toward the RE for patients showing better speech scores on the RE *versus* the LE (*r* > 0.48, *p* < 0.0002, *p*_*adj*_< 0.002, for word, syllabic, and phonemic scores recorded on the first block, and *r* > 0.34, *p* = 0.008, *p*_*adj*_ = 0.03, for speech scores recorded on the second block). There was no significant correlation between this ear preference (average of both blocks) and the HT asymmetry (*r* = 0.012, *p* = ns) and the SRT asymmetry (*r* = −0.03, *p* = ns), nor the 100% speech threshold asymmetry (*r* = 0.023, *p* = ns), nor the LI (*r* = −0.03, *p* = ns) or the HI (rho = 0.18, *p* = ns).

The self-reported listening effort increased significantly as word scores decreased, for both block 1 (*r* = −0.38, *p* < 0.003, *p*_*adj*_ < 0.02) and block 2 (*r* = −0.35, *p* < 0.006, *p*_*adj*_ < 0.03). It tended to increase as CI decreased (*r* = −0.29, *p* = 0.02, *p*_*adj*_ = 0.07). However, the cognitive status, assessed by the MOCA, was not correlated to the importance of CI (*r* = 0.02, *p* = ns).

### Age Effects

We did not observe any significant relationship between age and the MOCA score (*r* = −0.25, *p* = 0.06), the lexical test score, the LI (rho = 0.10, *p* = ns), the HT asymmetry (rho = 0.07, *p* = ns), and the better ear HT (rho = 0.22, *p* = 0.08), and the only weak correlations were observed between age and RE SRT (rho = 0.26, *p* < 0.05, *p*_*adj*_ = ns) (*r* = 0.16, *p* = ns for LE SRT).

We did not observe a significant modification of the LI with age, but the speech scores at the DLT decreased significantly as age (*r* = −0.44, *p* < 0.0005, *p*_*adj*_ < 0.005) and HT (*r* = −0.35, *p* = 0.007, *p*_*adj*_ < 0.03) increased and increased significantly with the MOCA score (*r* = 0.38, *p* < 0.003, *p*_*adj*_ < 0.02) (Table 1).

However, CI asymmetry decreased significantly as age increased (*r* = −0.33, *p* = 0.005, *p*_*adj*_ = 0.04, Figure 3D), which was due to a tendency for an increase of the LE CI with age (*r* = 0.28, *p* = 0.03, *p*_*adj*_ = ns), without any significant influence on the RE CI (*r* = −0.04, *p* = ns) (*z* = 1.42, *p* = ns). This impact was observed for the first block only, with a weak increase in word speech scores as a function of age for the LE (*r* = 0.31, *p* < 0.02, *p*_*adj*_ = 0.055). This relationship between age and CI asymmetry was significantly greater than the relationship between age and LI (*r* = −0.33 *versus r* = 0.04, *z* = 2.8, *p* = 0.005).

The improvement in percent scores from the first to the second block was not statistically different between the RE and LE. However, it decreased significantly as age increased (*r* = −0.38, *p* < 0.003, *p*_*adj*_ < 0.02), which was mainly due to a decrease of the improvement with age for the LE (*r* = −0.47, *p* < 0.0002, *p*_*adj*_ = 0.001), which was significantly stronger than the relationship with the RE (*r* = 0.08, *p* = ns; z = 2.7, *p* < 0.007). Age was the only statistically significant predictor of this improvement in scores from the first to the second block (the cognitive score, lexical test scores, and HT were not significant predictors).

## Discussion

This study shows a REA for CI on speech perceived in quiet in hearing-impaired patients, in the absence of a contralateral competing stimulus. Furthermore, this REA for top–down CI was significantly correlated to hemispheric asymmetry as measured by the DLT: the bigger the REA at the DLT, the greater the CI on the RE comparatively to the LE. This asymmetry in CI did not correlate with HT asymmetry nor speech threshold asymmetry, which argues against the involvement of peripheral asymmetry in the effect seen. The fact that this CI asymmetry was obtained by comparing RE and LE performances of the same individuals, in a simple word perception task performed in quiet and in the absence of competing stimulus in the contralateral ear, suggests that this asymmetry is more linked to the dominance of the left hemisphere for speech and verbal processing than to an attentional focus on the RE. Indeed, models explaining the REA obtained in DLT combine several elements: (1) dominance of the left hemisphere for verbal processing combined with more efficient crossed afferent pathways (than ipsilateral) to convey information, favoring the RE—left hemisphere path, (2) corpus callosum rate of information transfer between both hemispheres, and (3) top–down attention processes, allowing a focus on the relevant signal and inhibiting the contralateral signal. In the present study, there were no competing stimuli in the contralateral ear, so that differential attentional focus on one ear or the other is less likely to play a role in the observed REA. Furthermore, a significantly greater RE_CI than LE_CI was obtained, i.e., on the completion process that allows generating a meaningful percept from patchy afferent information.

One of the limitations of the study is the significant correlation obtained between HT asymmetry and the LI at the DLT. Admittedly, the DLT is very sensitive to differences in audibility between both ears (Tallus et al., 2007; Hugdahl et al., 2008), especially in hearing-impaired patients in whom both ears are rarely equivalent in peripheral deficits. By placing the intensity well above the 100% intelligibility threshold of the worse ear, we tried to counteract most of the hearing asymmetry, but this was not sufficient for the patients with the greatest HT asymmetry. However, as no significant correlation was obtained between CI asymmetry and HT asymmetry and as the correlation between CI asymmetry and LI was significantly stronger than the correlation with HT asymmetry, the REA observed here in CI cannot be attributed to HT asymmetry alone. A greater top–down CI on RE than on LE, linked to hemispheric laterality, could explain the superiority of speech perception scores obtained in clinical studies on the RE *versus* the LE on patients with single-sided deafness (Wettstein and Probst, 2018) or in patients with cochlear implants (Henkin et al., 2008). Indeed, this superiority of the RE CI over the LE is maintained during a repetition of the same task (the second block), which shows a general increase in CI, which can be explained by memory and lexical selection: a single presentation of 60 words was sufficient to facilitate the perception of those same 60 words after a short pause, although the patients were not warned of the presence of the same words in the two blocks. This facilitation, shown by an increase of 4.7% in percent correct score between the two repetitions, can be attributed to enhanced activation of the relevant lexicon, which was reduced in size (from all disyllabic nouns in the French language down to 60) thanks to the previous presentation of the words. This effect was linked to an increase in self-assessed effort for the second block *versus* the first one but was not significantly linked to more cognitive factors such as the MOCA test or the lexical test. Both ears showed an improvement in scores from the first to the second block, with no statistically significant difference between them. However, we observed a significantly stronger decrease with age, of this improvement between both blocks, for LE scores *versus* RE scores. This can be attributed to the stronger link of LE CI with age observed for the first block, with more reliance on top–down mechanisms to repeat a correct word when the latter is presented to an “old” LE than to a younger LE. This age effect could be attributed, at least partly, to the linguistic material used: French disyllabic word lists designed in the fifties, which contains more familiar words to the elderly than to the younger population (Moulin and Richard, 2015), allowing better scores in the most elderly patients *via* greater possibilities of using CI to compensate peripheral signal degradation. Using different degrees in speech in noise degradation, several studies have pointed out maintenance of top–down restoration processes with age and even better restoration processes in older populations with mild hearing-losses as a way of compensating signal degradation, perhaps helped by greater lexical knowledge in the elderly (Zekveld et al., 2011; Benichov et al., 2012; Saija et al., 2014). The differential age effect observed here, i.e., stronger on the LE *versus* the RE of the same individuals, could be attributed to the generally weaker functional status of the LE *versus* the RE. Indeed, the LE has been shown to have cochlear responses of lower amplitude (Keefe et al., 2008) and to be more sensitive to noise-induced degradation (Job et al., 1998; Nageris et al., 2007; Le et al., 2017) and to the influence of age (Qian et al., 2021). This greater degradation of the LE with age could engender greater top–down compensation for older patients compared to younger ones, visible here as a growing LECI with age. This yielded a significant decrease of CI asymmetry with age, with no significant modification of LI with age. Several studies have shown a shift of lateralization, from the dominant left hemisphere toward the right hemisphere, as the challenge is increased in speech in noise perception tasks in normal-hearing individuals (Shtyrov et al., 1999; Bidelman and Howell, 2016). This shift toward less asymmetry has also been observed in older listeners comparatively to younger ones, in speech in noise perception tasks (Zan et al., 2020), and in temporal auditory processing (Farahani et al., 2020). Although the speech perception task here involves perception in quiet, it is still extremely challenging for the participants, as the intensities were adjusted to have perception scores around 50%, and as all participants are hearing-impaired. As the CI in the present study depends mainly on the syllables perception (as both CI_*pho_word*_ and CI_*syll_word*_ were highly correlated), and as the phonetic details perception to reconstruct syllables have been shown to be similar in quiet and in noise in hearing-impaired hearing-aided listeners (Miller et al., 2017), the present results could be extrapolated to speech in noise perception. Here, we obtained a decrease of CI asymmetry with age, in hearing-impaired listeners, in a speech perception task in quiet, similar to previous studies in speech in noise perception, in agreement with the HAROLD model of reduced asymmetry with age (Cabeza, 2002; Dolcos et al., 2002).

Interestingly, the same LE that showed an increase in speech perception scores with age in the main perception task, in relationship with an increase in LECI with age, showed a significant decrease in scores with age for the DLT (similarly to the RE). This can be attributed to the differences in tasks: the main task does not involve any competing stimulus, i.e., less attentional mechanisms and inhibition, but involves strong top–down processes, as the task is performed at low intensity. Conversely, the DLT involves strong attentional processes (inhibiting one ear and focusing on the other), heavy short-term memory load, but less top–down CI, as the words are presented at comfortable levels (i.e., can be easily perceived) and are monosyllabic words, therefore involving less CI than the disyllabic words. The difference in scores between the main task (around 55%) and the DLT (around 30%), performed at a much greater intensity than the main task, highlights the toll that the DLT takes on the elderly hearing-impaired patients. In addition, the DLT performance scores increased significantly with the MOCA score, highlighting the strong dependence of the DLT on cognitive status (Hugdahl et al., 2009; Bouma and Gootjes, 2011).

## Conclusion

Greater contextual influence, at the word level, during speech perception in quiet, was observed in the RE *versus* the LE of hearing-impaired patients. This asymmetry of CI was linked to hemispheric asymmetry and decreased significantly with age, in agreement with the right-shift in hemispheric asymmetry linked to speech in noise perception in older people.

## Data Availability Statement

The datasets presented in this article are not readily available outside the European Union, because it was collected within the framework of the General Data Protection Regulation (GDPR), which is the new legislation that governs the processing of personal data in Europe. The participants did not give their consent for transmission of their indirectly identifying health data outside the European Union. Therefore, we cannot provide individual health data, that are indirectly identifying and belong to the participants. Parts of the dataset that are not indirectly nor directly identifying, are made available upon request to the author. Requests to access the datasets should be directed to AM, annie.moulin@cnrs.fr.

## Ethics Statement

The studies involving human participants were reviewed and approved by “comité de protection des personnes” de Ile de France II, France”, n°21.01.08.67105//4314802. The patients/participants provided their written informed consent to participate in this study.

## Author Contributions

AM designed the study, built the specific materials used, analyzed the data, did the statistics and illustrations, and wrote the manuscript.

## Conflict of Interest

The author declares that the research was conducted in the absence of any commercial or financial relationships that could be construed as a potential conflict of interest.

## Publisher’s Note

All claims expressed in this article are solely those of the authors and do not necessarily represent those of their affiliated organizations, or those of the publisher, the editors and the reviewers. Any product that may be evaluated in this article, or claim that may be made by its manufacturer, is not guaranteed or endorsed by the publisher.

## References

[B1] Benichov J. Cox L. C. Tun P. A. Wingfield A. (2012). Word recognition within a linguistic context: effects of age, hearing acuity, verbal ability and cognitive function. *Ear Hear.* 32 250–256. 10.1097/AUD.0b013e31822f680f PMC3253325 21918453

[B2] Benjamini Y. Hochberg Y. (1995). Controlling the false discovery rate: a practical and powerful approach to multiple testing. *J. R. Stat. Soc. Ser. B* 57 289–300.

[B3] Bidelman G. M. Howell M. (2016). Functional changes in inter- and intra-hemispheric cortical processing underlying degraded speech perception. *Neuroimage* 124 581–590. 10.1016/j.neuroimage.2015.09.020 26386346

[B4] Boothroyd A. Nittrouer S. (1988). Mathematical treatment of context effects in phoneme and word recognition. *J. Acoust. Soc. Am.* 84 101–114. 3411038 10.1121/1.396976

[B5] Bouma A. Gootjes L. (2011). Effects of attention on dichotic listening in elderly and patients with dementia of the Alzheimer type. *Brain Cogn.* 76 286–293. 10.1016/j.bandc.2011.02.008 21429649

[B6] Cabeza R. (2002). Hemispheric asymmetry reduction in older adults: the HAROLD model. *Psychol. Aging* 17 85–100. 10.1037//0882-7974.17.1.85 11931290

[B7] Davis M. H. Johnsrude I. S. (2007). Hearing speech sounds: top-down influences on the interface between audition and speech perception. *Hear. Res.* 229 132–147. 10.1016/j.heares.2007.01.014 17317056

[B8] Deltour J. (1998). *Echelle De Vocabulaire De Mill-Hill (traduction française).* Paris: Éditions et Applications Psychologiques.

[B9] Demanez L. Demanez J. P. (2011). Le bilan auditif central et les troubles auditifs centraux chez les jeunes enfants. *Cah. L Audit.* 24 30–35.

[B10] Dolcos F. Rice H. J. Cabeza R. (2002). Hemispheric asymmetry and aging: right hemisphere decline or asymmetry reduction. *Neurosci. Biobehav. Rev.* 26 819–825. 12470693 10.1016/s0149-7634(02)00068-4

[B11] Farahani E. D. Wouters J. van Wieringen A. (2020). Neural generators underlying temporal envelope processing show altered responses and hemispheric asymmetry across age. *Front. Aging Neurosci.* 12:596551. 10.3389/fnagi.2020.596551 33343335 PMC7746817

[B12] Fournier J.-E. (1951). *Audiométrie Vocale: Les Épreuves D’intelligibilité et Leurs Applications Au Diagnostic, À l’expertise et À la Correction Prothétique Des surdités.* Paris: Maloine.

[B13] Geffen G. Quinn K. (1984). Hemispheric specialization and ear advantages in processing speech. *Psychol. Bull.* 96 273–291. 10.1037/0033-2909.96.2.273 6385045

[B14] Gordon-Salant S. Fitzgibbons P. J. Yeni-Komshian G. H. (2011). Auditory temporal processing and aging: implications for speech understanding of older people. *Audiol. Res.* 1:e4. 10.4081/audiores.2011.e4 PMC4627162 26557313

[B15] Hartvig Jensen J. Johansen P. A. Børre S. (1989). Unilateral sensorineural hearing loss in children and auditory performance with respect to right/left ear differences. *Br. J. Audiol.* 23 207–213. 10.3109/03005368909076501 2790305

[B16] Henkin Y. Taitelbaum-Swead R. Hildesheimer M. Migirov L. Kronenberg J. Kishon-Rabin L. (2008). Is there a right cochlear implant advantage? *Otol. Neurotol.* 29 489–494. 10.1097/MAO.0b013e31816fd6e5 18401283

[B17] Hiscock M. Kinsbourne M. (2011). Attention and the right-ear advantage: What is the connection? *Brain Cogn.* 76 263–275. 10.1016/j.bandc.2011.03.016 21507543

[B18] Hugdahl K. Westerhausen R. Alho K. Medvedev S. Hämäläinen H. (2008). The effect of stimulus intensity on the right ear advantage in dichotic listening. *Neurosci. Lett.* 431 90–94. 10.1016/j.neulet.2007.11.046 18162310

[B19] Hugdahl K. Westerhausen R. Alho K. Medvedev S. Laine M. Hämäläinen H. (2009). Attention and cognitive control: unfolding the dichotic listening story. *Scand. J. Psychol.* 50 11–22. 10.1111/j.1467-9450.2008.00676.x 18705670

[B20] Job A. Grateau P. Picard J. (1998). Intrinsic differences in hearing performances between ears revealed by the asymmetrical shooting posture in the army. *Hear. Res.* 122 119–124. 9714580 10.1016/s0378-5955(98)00104-x

[B21] Keefe D. H. Gorga M. P. Jesteadt W. Smith L. M. (2008). Ear asymmetries in middle-ear, cochlear, and brainstem responses in human infants. *J. Acoust. Soc. Am.* 123 1504–1512. 10.1121/1.2832615 18345839 PMC2493569

[B22] Kimura D. (1961). Cerebral dominance and the perception of verbal stimuli. *Can. J. Psychol.* 15 166–171. 10.1037/h0083219

[B23] Kinsbourne M. (1970). The cerebral basis of lateral asymmetries in attention. *Acta Psychol.* 33 193–201. 10.1016/0001-6918(70)90132-0 5445964

[B24] Le T. N. Straatman L. V. Lea J. Westerberg B. (2017). Current insights in noise-induced hearing loss: a literature review of the underlying mechanism, pathophysiology, asymmetry, and management options. *J. Otolaryngol. Head Neck Surg.* 46:41. 10.1186/s40463-017-0219-x 28535812 PMC5442866

[B25] Miller J. D. Watson C. S. Leek M. R. Dubno J. R. Wark D. J. Souza P. E. (2017). Syllable-constituent perception by hearing-aid users: common factors in quiet and noise. *J. Acoust. Soc. Am.* 141 2933–2946. 10.1121/1.4979703 28464618 PMC5848866

[B26] Moulin A. Richard C. (2015). Lexical influences on spoken spondaic word recognition in hearing-impaired patients. *Front. Neurosci.* 9:476. 10.3389/fnins.2015.00476 26778945 PMC4688363

[B27] Nageris B. I. Raveh E. Zilberberg M. Attias J. (2007). Asymmetry in noise-induced hearing loss: relevance of acoustic reflex and left or right handedness. *Otol. Neurotol.* 28 434–437. 10.1097/mao.0b013e3180430191 17435523

[B28] Nasreddine Z. S. Phillips N. A. Bédirian V. Charbonneau S. Whitehead V. Collin I. (2005). The montreal cognitive assessment, MoCA: a brief screening tool for mild cognitive impairment. *J. Am. Geriat. Soc.* 53 695–699. 15817019 10.1111/j.1532-5415.2005.53221.x

[B29] Oldfield R. C. (1971). The assessment and analysis of handedness: the Edinburgh inventory. *Neuropsychologia* 9 97–113. 5146491 10.1016/0028-3932(71)90067-4

[B30] Phillips S. L. Gordon-Salant S. Fitzgibbons P. J. Yeni-Komshian G. (2000). Frequency and temporal resolution in elderly listeners with good and poor word recognition. *J. Speech Lang. Hear. Res.* 43 217–228. 10.1044/jslhr.4301.217 10668664

[B31] Pichora-Fuller K. M. (2008). Use of supportive context by younger and older adult listeners: balancing bottom-up and top-down information processing. *Int. J. Audiol.* 47 S72–S82. 19012114 10.1080/14992020802307404

[B32] Qian M. Wang Q. Yang L. Wang Z. Hu D. Li B. (2021). The effects of aging on peripheral and central auditory function in adults with normal hearing. *Am. J. Transl. Res.* 13 549–564. 33594309 PMC7868840

[B33] Saija J. D. Akyürek E. G. Andringa T. C. Başkent D. (2014). Perceptual restoration of degraded speech is preserved with advancing age. *J. Assoc. Res. Otolaryngol.* 15 139–148. 10.1007/s10162-013-0422-z 24198087 PMC3901857

[B34] Shtyrov Y. Kujala T. Ilmoniemi R. J. Näätänen R. (1999). Noise affects speech-signal processing differently in the cerebral hemispheres. *Neuroreport* 10 2189–2192. 10424696 10.1097/00001756-199907130-00034

[B35] Steiger J. H. (1980). Tests for comparing elements of a correlation matrix. *Psychol. Bull.* 87 245–251.

[B36] Tallus J. Hugdahl K. Alho K. Medvedev S. Hämäläinen H. (2007). Interaural intensity difference and ear advantage in listening to dichotic consonant–vowel syllable pairs. *Brain Res.* 1185 195–200. 10.1016/j.brainres.2007.09.012 17920571

[B37] Wettstein V. G. Probst R. (2018). Right ear advantage of speech audiometry in single-sided deafness. *Otol. Neurotol.* 39 417–421. 10.1097/MAO.0000000000001756 29533329 PMC5882291

[B38] Zan P. Presacco A. Anderson S. Simon J. Z. (2020). Exaggerated cortical representation of speech in older listeners: mutual information analysis. *J. Neurophysiol.* 124 1152–1164. 10.1152/jn.00002.2020 32877288 PMC7717162

[B39] Zekveld A. A. Kramer S. E. Festen J. M. (2011). Cognitive load during speech perception in noise: the influence of age, hearing loss, and cognition on the pupil response. *Ear Hear.* 32 498–510. 21233711 10.1097/AUD.0b013e31820512bb

