# Supplementary material for: Ear Asymmetry and Contextual Influences on Speech Perception in Hearing-Impaired Patients
Source: Front Neurosci. 2022 Mar 18;16:801699. doi: 10.3389/fnins.2022.801699 (PMC8974937; doi:10.3389/fnins.2022.801699)
Supplement: Supplementary file 1 [file Data_Sheet_1.pdf]

Supplementary material

Moulin A. (2022) Ear Asymmetry and Contextual Influences on Speech Perception in Hearing-Impaired Patients.
Frontiers in Neuroscience, https://doi.org/10.3389/fnins.2022.801699

1. Material and methods

Detailed Dichotic Listening Test (DLT) procedure

A validated French language dichotic listening test (Demanez, L and Demanez, JP, 2011) was performed at an intensity of at least 6 dB above the 100% speech perception threshold of the worse ear, using 2 blocks of 12 trials, each trial composed of 2 pairs of 2 monosyllabic words: 2 meaningful monosyllabic words were presented simultaneously to the RE and LE (i.e., a total of 4 words), followed by a silent gap of 5.2 seconds, before the next trial. The task was performed twice, with the stimuli in a randomized order, and right and left side inverted for the second block, in agreement with the test manual. A free recall paradigm was used: the participants were invited to repeat all the words heard (i.e., a maximum of 4 words). Prior to the test, a training session involving 3 trials was used, different from the words used for the test, to make sure the task was well understood by the patient and to check the appropriateness of the intensities chosen. None of the words of the dichotic test appeared in the other speech perception tests performed.

The DLT performance scores showed an average of 30.4 (SD=11.44), median at 27.6, which corresponds to the normative data established for 65 to 74 years old by the test creators (Demanez, L and Demanez, JP, 2011).

2. Results

2.1. Population characteristics

*** Age and hearing loss analysis per gender** didn't reveal any significant differences.

No significant difference in age was observed between the 29 men (70.9, SD=10.3) and the 31 women participants (67.8, SD=14.8) $F(1,58)=0.85$, p=ns.

No significant difference in better ear hearing thresholds [35.3 dB HL (SD=12.7) for women versus 31.1, SD=11.8 for men, $F(1,58)=1.8$, p=ns], nor in worse ear hearing thresholds [41.2 dB HL (SD=14) for women versus 38.2, SD=12.7 for men, $F(1,58)=0.8$, p=ns] were obtained between men and women.

*** DLT scores as a function of age.**

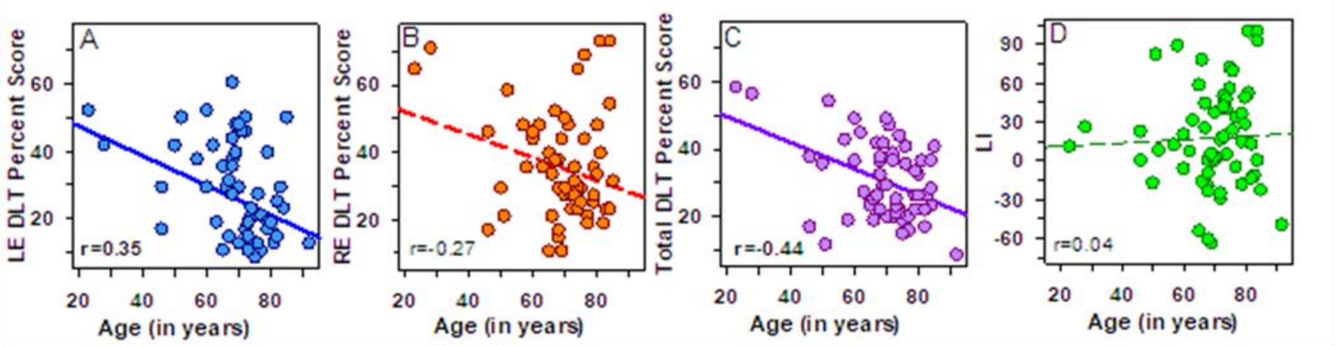

Supplemental figure 1: *DLT scores as a function of age. The scores in percentage obtained for the DLT, are shown as a function of age, for the left ear (fig. A), for the right ear (fig. B), for both ears combined (fig. C). The laterality index is shown as a function of age. Significant correlation coefficients are shown in bold (i.e. |r|>0.30), with continuous regression lines, where non-significant correlations are shown in plain fonts with dashed regression lines.*

2.2. Distributions of raw j factors.

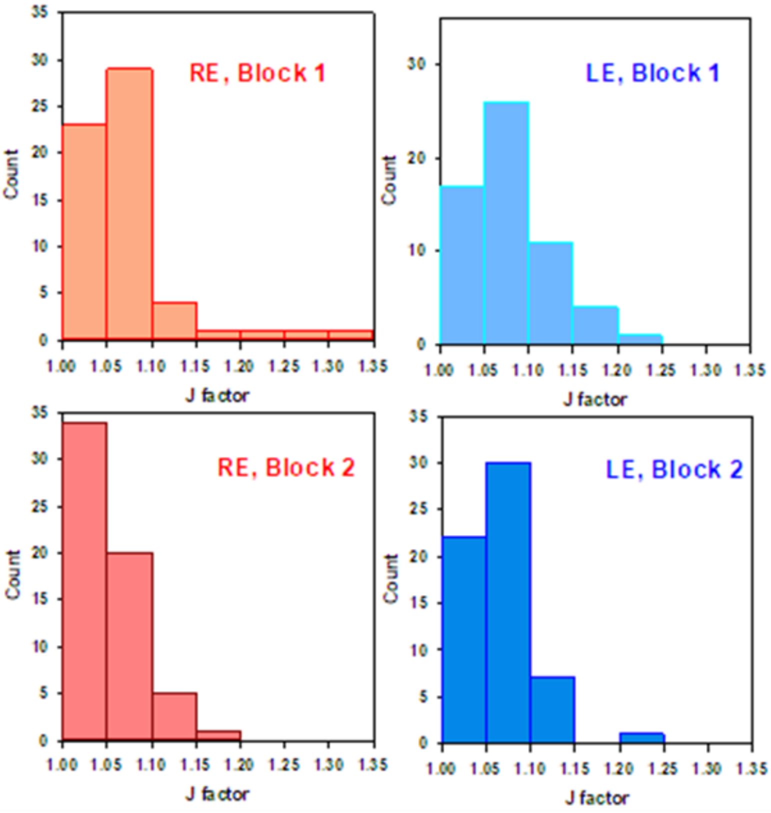

Supplemental figure 2: *Distribution of j factors obtained in the right ear (red histograms), and in the left ear (blue histogram), for the first block (top figures) and the second blok (bottom figures), in 60 hearing-impaired participants. Contextual influences increase as the j factor gets closer to 1.0.*

2.3. Contextual influence (calculated using syllabic and word scores) (CI*syll_word)*

Contextual influences asymmetry

CI*syll_word* was greater in the RE versus the LE (F(1,59)=8.1, p<0.006, η_p=0.12, ω^2 = 0.013), and for the second block versus the first one (F(1,59)=22.8, p<0.00001, η_p=0.28, ω^2 = 0.036), without any significant interaction between the two. Type of list, nor hearing threshold asymmetry, used as covariates, yielded any significant interaction. However, there was a small, but statistically significant interaction with the laterality index: Ear x Block x LI (F(1,58)=4.3, p<0.05, η_p=0.07, ω^2 = 0.009). When the LI was used as covariate, the main Ear effect became non statistically significant (F(1,58)=3.95, p=0.05), but the significant block effect remained.

Contextual influences asymmetry and laterality indexes

The CI*syll_word* asymmetry increased significantly with the LI (rho=0.34, p<0.01 for CI*syll_word*), only for the first block (rho=-0.09, *p*=ns for the second block). The correlations obtained in the first block were significantly greater than the ones obtained in the second block (z=2.9, *p*<0.004). A tendency for a correlation between CI asymmetry and LI was still obtained when averaging the CI asymmetry of both blocks (rho=0.23, *p*<0.07).

A multi-regression model showed that the CI*syll_words* asymmetry, was explained significantly by the LI (β=0.31, p<0.01), and age (β =-0.39, p<0.003) (r^2= 0.24, F(2,57)=8.8, p<0.0005) : it increased with LI, and decreased with age. None of the other tested variables (in particular HT asymmetry, cognitive tests, handedness, HT) reached statistical significance.

2.4. Contextual influence calculated using phonemes and syllabic scores (CI*pho_syll)*

CI calculated using phonemes and syllables was constantly and significantly lower to the other CI indexes (mean 1.01, SD=0.03 versus 1.05, SD=0.05 for CI*syll_word*, W=248, p<10^{-10}). This is expected as phonemes and syllabic scores are much closer to one another, and most of the contextual influence is captured from syllabic to word scores. There is much less contextual influence allowing to complete a syllable, which doesn't involve much semantic content, from only phonemes. The statistical results concerning CI*pho_syll* are much weaker (not statistically significant) than the other indices. In the literature, CI*pho_syll* are generally not calculated.

When CI was analysed by taking into account phonemes and syllables, no statistically significant ear effect was found F(1,59)=0.5, and a weak significant block effect was obtained, with greater CI for the second block versus the first one F(1,59)=4.2, p<0.05, ηp=0.07, ω = 0). t=2.05, Cohen's d=0.26, p_{bonf}<0.05).

-=-=-=-=-=-=-
